# Exploratory assessment of bleeding risk associated with concurrent use of Anti-VEGF agents and anticoagulants in oncology

**DOI:** 10.3389/fphar.2026.1759971

**Published:** 2026-04-08

**Authors:** Dehua Liao, Yang Zhao, Yuanxiang Jin, Minghui Long, Shengfeng Wang, Shanshan Chen

**Affiliations:** 1 Department of Pharmacy, Hunan Cancer Hospital/The Affiliated Cancer Hospital of Xiangya School of Medicine, Central South University, Changsha, Hunan, China; 2 Department of Interventional Radiology and Vascular Surgery, Hunan Cancer Hospital, The Affiliated Cancer Hospital of Xiangya School of Medicine, Central South University, Changsha, Hunan, China; 3 Department of Clinical Pharmacy, Hunan University of Medicine General Hospital, Huaihua, Hunan, China; 4 Department of Pharmacy, The Third Xiangya Hospital, Central South University, Changsha, Hunan, China

**Keywords:** anticoagulant, bevacizumab, drug-drug interaction, hemorrhage, tyrosine kinase inhibitor

## Abstract

**Background:**

The concurrent use of anti-vascular endothelial growth factor (VEGF) agents and anticoagulants has become increasingly unavoidable in oncology, while the associated bleeding risk remains poorly characterized.

**Methods:**

A retrospective analysis combined with pharmacovigilance assessment was conducted to assess the associated bleeding risk. Patients who received concurrent use of anti-VEGF agents and anticoagulants at Hunan Cancer Hospital between 1 January 2010, and 1 October 2025, were included. Data on demographics, safety and duration of co-administration were collected. Additionally, drug-drug interaction signals were analyzed using FDA Adverse Event Reporting System (FAERS) (Q1, 2013 - Q4, 2024) via Ω shrinkage measure.

**Results:**

Among 208 patients (83 on TKIs, 125 on Bev), 32.53% of TKI users and 41.6% of Bev users received anticoagulants for VTE treatment. In the TKI/Bev VTE treatment groups, 51.85% and 61.54% of patients were administered prophylactic anticoagulants, respectively. Rivaroxaban was the primary anticoagulant, followed by low molecular weight heparin (LMWH). For TKIs, the median co-administration duration was 4 days in the prophylaxis group and 30 days in the treatment group, whereas for Bev, the corresponding duration was 28.5 days and 50 days, respectively. Bleeding events were rare across all groups (<6%), with no grade ≥3 events observed. In FAERS, only a potential bleeding signal was observed for Bev-LMWH co-administration (Ω_025_ = 0.06).

**Conclusion:**

TKI-DOAC/LMWH and Bev-DOAC co-administration appeared to be safe, especially with short-term, prophylactic anticoagulant dosage. Given a potential bleeding signal, Bev-LMWH co-administration requires rigorous clinical monitoring. Owing to the hypothesis-generating nature of our study, definitive safety or causality require prospective validation.

## Graphical Abstract

**Figure:**
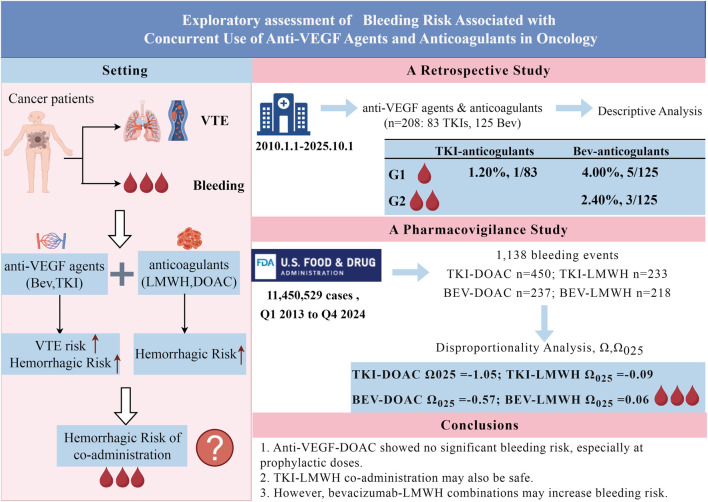

## 1 Introduction

Vascular endothelial growth factor (VEGF) is a key signaling protein essential for embryonic development and tissue repair. In the tumor microenvironment, hypoxia induces significant expression of VEGF in tumor and stromal cells, which drives angiogenesis, increases vascular permeability, and promotes tumor growth, invasion, and metastasis (Ghalehbandi et al., 2023). In this respect, anti-VEGF drugs, primarily including bevacizumab (Bev) and tyrosine kinase inhibitors (TKIs), have been developed and approved for clinical use (Patel et al., 2023). These two classes of anti-VEGF drugs provide undeniable survival advantages in a variety of malignant tumors. Additionally, a growing body of clinical trial evidence has indicated that combinations of anti-VEGF agents, chemotherapy, and/or immune checkpoint inhibitors exert synergistic anti-tumor effects (Bodor et al., 2023; Cheng et al., 2022; Llovet et al., 2023), thus further expanding the application of anti-VEGF agents. Despite demonstrated clinical benefits, Bev and TKIs are associated with an increased risk of vascular complications including bleeding and thrombosis, as inhibition of VEGF pathway disrupts endothelial function and decreases vascular integrity (Je et al., 2009).

Patients with cancer are at a high risk to develop venous thromboembolism (VTE) due to the hypercoagulable state, compression on the blood vessels by the tumor, and bedridden status (Mulder et al., 2021; Ohashi et al., 2020). VTE ranks as the second leading cause of cancer-related death, underscoring the critical role of anticoagulants in the prophylaxis and treatment of VTE (Khorana et al., 2007). Low molecular weight heparins (LMWH) are the cornerstone of cancer-associated VTE in the past decade. However, direct oral anticoagulants (DOACs) have quickly become attractive alternatives since approval due to their non-inferiority compared to LMWH, convenient oral administration, and predictable pharmacokinetics without routine monitoring (Schrag et al., 2023). Additionally, advancements in cancer treatment have prolonged the survival of patients, while increase the risk of cardiovascular events requiring anticoagulation such as atrial fibrillation (AF) and stroke (Yasui and Fujita, 2022; Navi et al., 2021). DOACs have now become the first-line anticoagulant options to prevent these events (Van Gelder et al., 2024; Bushnell et al., 2024).

The concurrent use of anti-VEGF agents and anticoagulants may become increasingly common in cancer patients, driven by the expanding use of anti-VEGF agents and the need for routine anticoagulation. Moreover, cancer patients demonstrate an elevated risk of bleeding due to local tumor invasion and regression, pathological tumor vasculature, and tumor-associated coagulopathy (Englis et al., 2024), which further raises safety concerns associated with this combination. The existing randomized controlled trials of anti-VEGF agents have tended to exclude patients on anticoagulants. Hence, current evidence on the bleeding risk of this combination is limited and heterogeneous. A retrospective analysis of small sample size (n = 7) supported the safety of Bev-DOACs combination in patients with ovarian cancer (Komiyama et al., 2019). However, a major bleeding event was reported in a patient with metastatic renal cell carcinoma (RCC) who received a combination of cabozantinib and rivaroxaban (Chen et al., 2021). This study aims to conduct a real-world evidence (RWE) analysis using a single-center retrospective dataset in conjunction with the FAERS database, thereby providing evidence for the safety of the concurrent use of anti-VEGF agents and anticoagulants.

## 2 Methods

### 2.1 A retrospective analysis

#### 2.1.1 Study population

**FIGURE 1:**
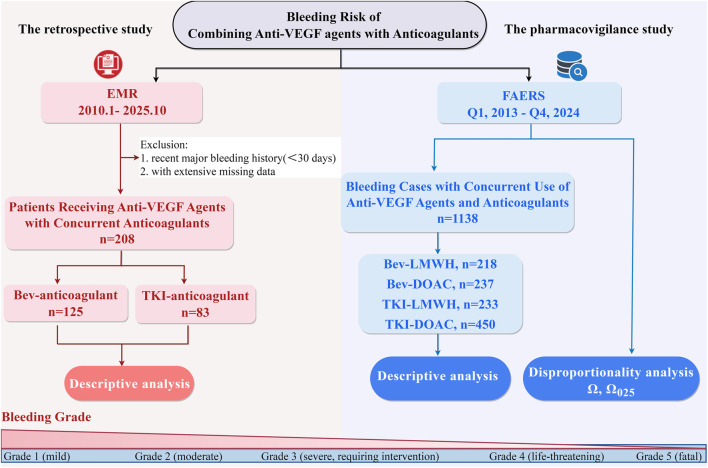
The flow diagram of the retrospective and pharmacovigilance analysis. The retrospective analysis included patients at Hunan Cancer Hospital who concurrently received anti-VEGF agents and anticoagulants from 1 January 2010, to 1 October 2025. DDI signals between these agents were analyzed using FAERS data (Q1, 2013 - Q4, 2024) via Ω shrinkage measure. Bleeding events were described and graded according to CTCAE v5.0 based on different combinations of these agents.

Patients who were diagnosed with solid cancer and concurrently treated with anti-VEGF agents and anticoagulants at Hunan Cancer Hospital from 1 January 2010 to 1 October 2025 were included (Figure 1). Anti-VEGF agents were defined as Bev or TKIs (e.g., apatinib, lenvatinib, regorafenib). Anticoagulants comprised DOACs and LMWH. Anticoagulant dosages were categorized as prophylactic, intermediate, or therapeutic, as specified in Supplementary Table S1. Patients with a major bleeding event within the preceding 30 days or extensive missing data were excluded. Clinical data was extracted from electronic medical records (EMR), including cancer type, patient demographic, details of anti-VEGF agents and anticoagulants, details of thrombotic events and bleeding complications.

The “concurrent use” was defined as the co-administration of anti-VEGF agents and anticoagulants with any overlap. The detailed definitions and calculation methods for the duration of co-administration were as follows: most TKIs were prescribed for daily continuous dosing, while a small subset involved scheduled treatment interruptions. Therefore, the duration of TKI-anticoagulant co-administration was defined as the interval from co-administration initiation to discontinuation, including all scheduled treatment interruptions. Bev was typically administered every 2 or 3 weeks with a half-life of approximately 20 days. The duration of Bev-anticoagulant co-administration was defined as the number of days that anticoagulants were administered during the Bev treatment cycle(s). A schematic diagram depicting the exposure timeline is provided in Supplementary Figure S1.

#### 2.1.2 Assessment of bleeding events

Bleeding events were graded according to Common Terminology Criteria for Adverse Events (CTCAE) v5.0, as follows: Grade 1 (mild), Grade 2 (moderate), Grade 3 (severe, requiring intervention), Grade 4 (life-threatening), and Grade 5 (fatal). For each bleeding event, data were collected on the bleeding site, date of occurrence, grade, and any therapeutic interventions administered.

#### 2.1.3 The outcomes of thromboembolic events

The progression of thrombosis was evaluated utilizing ultrasonography or computed tomography (CT). The outcomes of thromboembolic events were categorized into four categories: improved, unchanged, aggravated, and unevaluated.

### 2.2 A pharmacovigilance study

#### 2.2.1 Data source and processing

FAERS is an established surveillance tool that enables signal detection and quantitative assessment of drug-adverse events. A pharmacovigilance analysis was performed using data spanning from Q1 2013 to Q4 2024, with deduplication procedures implemented as FDA recommended (Figure 1). For cases with identical CASEID, the most recent report based on FDA_DT was retained; when both CASEID and FDA_DT matched, the highest PRIMARYID was selected. This process yielded a final analytical data of 11,450,529 unique reports.

In the absence of a standardized coding system for drug names in FAERS, both generic and brand names of anti-VEGF agents and anticoagulants were employed (Supplementary Table S2). Adverse event (AE) reports in FAERS were coded using preferred terms (PTs) from the Medical Dictionary for Regulatory Activities (MedDRA). All bleeding-related AEs in the study were coded in PTs according to MedDRA version 28.0. Additionally, various PTs were integrated to characterize a specific clinical syndrome via an algorithmic methodology referred to Standardized MedDRA Queries (SMQs). A total of 521 PTs categorized within “hemorrhage” SMQ (Supplementary Table S3) were searched in FAERS. Data from “hemorrhage” reports associated with concurrent use of anti-VEGF agents and anticoagulants were systematically collected.

#### 2.2.2 Data mining algorithm

The Ω shrinkage method, recommended by World Health Organization Uppsala Monitoring Center and identified as the most conservative disproportionality algorithm in a prior study (Noguchi et al., 2020), was used to further quantify drug-drug interaction (DDI) signals. When Ω is positive, the concurrent use of two drugs confers a greater risk of a specific adverse event than the sum of the risks associated with their individual use (Noguchi et al., 2019). Hence, Ω_025_ > 0, representing a positive lower boundary of 95% CI, was utilized as a threshold to detect signals from the concurrent use of drug D1 and drug D2. In other words, it indicates the reporting frequency of specific drug-drug-event triplets in the dataset relative to the expected frequency based on the individual reporting rates of each drug. A significant signal was defined as Ω_025_ > 0, indicating elevated bleeding risk with concurrent use. The detailed methodology for calculating Ω was outlined in Supplementary Table S4.

### 2.3 Statistical analysis

Descriptive statistics were calculated for patient demographics. All continuous variables were summarized as median and inter-quartile (IQR), while categorical variables were presented as number and percentages. All analyses were performed using SAS version 9.4.

## 3 Results

### 3.1 A retrospective analysis

#### 3.1.1 Patient characteristics

In our study, a total of 208 patients were included, with 83 receiving TKIs and 125 receiving Bev, as outlined in Table 1. Among TKI users, 67.47% used anticoagulants for VTE prophylaxis and 32.53% for VTE treatment, while among Bev users, 58.40% used for prophylaxis and 41.6% for treatment. As shown in Supplementary Table S5, thrombotic events were diverse in anatomical site, with the majority presenting as acute events for the TKIs group. By contrast, distal/upper extremity deep vein thrombosis (DVT) was the predominant thrombotic event in the Bev group, and the majority of thrombotic events in this cohort were non-acute. Apatinib, lenvatinib, and regorafenib were the most commonly administered TKIs. The most frequent tumor types in the TKI-VTE prophylaxis and treatment groups were hepatocellular carcinoma, gynecological cancer, colorectal carcinoma, and gastric cancer. In contrast, colorectal cancer and gynecological cancer were the most frequent tumor types in the Bev-VTE prophylaxis and treatment groups, which were aligned with their respective clinical indications. In addition, the prevalence of high-risk baseline factors affecting bleeding risk (thrombocytopenia, abnormal renal/hepatic function, brain metastases, and concurrent use of antiplatelet agents or NSAIDs) was low across all cohorts, with details provided in Supplementary Table S6.

**TABLE 1:** Characteristics of patients on concurrent anti-VEGF agents and anticoagulants.

Parameters	TKI(n = 83)		Bev (n = 125)	
	VTE prophylaxis (n = 56)	VTE treatment (n = 27)	VTE prophylaxis (n = 73)	VTE treatment (n = 52)
Male, n (%)^a^	33 (58.93%)	16 (59.26%)	34 (46.58%)	18 (34.62%)
Median age (IQR), y	56 (50–60.5)	55 (51–60)	58 (53–66)	58 (51–65)
Cancer type, n (%)				
Liver	17 (30.36%)	11 (40.74%)	2 (2.74%)	2 (3.85%)
Colorectal	4 (7.14%)	6 (22.22%)	56 (76.71%)	21 (40.38%)
Gynecological	11 (19.64%)	5 (18.52%)	10 (13.70%)	19 (36.54%)
Gastric	6 (10.71%)	4 (14.81%)	1 (1.37%)	1 (1.92%)
Other	18 (32.15%)	1 (3.70%)	4 (5.48%)	9 (17.31%)
Types of TKIs, n (%)				
Apatinib	22 (39.29%)	12 (44.44%)	​	​
Lenvatinib	20 (35.71%)	9 (33.33%)	​	​
Regorafenib	6 (10.71%)	6 (22.22%)	​	​
Others	8 (14.28%)	​	​	​
Types of anticoagulants, n (%)				
LMWH	28 (50.00%)	5 (18.52%)	​	​
Rivaroxaban	25 (44.64%)	21 (77.78%)	67 (91.78%)	43 (82.69%)
LMWH plus rivaroxaban	3 (5.36%)	1 (3.70%)	6 (8.22%)	9 (17.31%)
Dosage of anticoagulants, n (%)				
Prophylactic	56 (100.00%)	14 (51.85%)	73 (100.00%)	32 (61.54%)
Intermediate	​	2 (7.41%)	​	​
Therapeutic	​	11 (40.74%)	​	20 (38.46%)
Duration of combined use				
Median duration (days,IQR)	4 (2.9–25)	30 (15–35)	28.5 (15–53.5)	50 (20–97)

^a^ All percentages were calculated relative to the respective column totals.

In the TKI-VTE prophylaxis group, rivaroxaban was used in 50% of cases, LMWH in 44.64%, and cases receiving the two drugs at different time points accounted for 5.36%. In the TKI-VTE treatment group, rivaroxaban was used in 77.78% of cases and LMWH in 18.52%. Notably, 51.85% of patients in this group received prophylactic-dose anticoagulants, while 40.74% were administered therapeutic doses. Rivaroxaban was also the most common anticoagulant used in combination with Bev, accounting for 91.78% and 82.69% of the Bev-VTE prophylaxis group and the treatment group. The rest were switched from short-term LMWH to rivaroxaban. In the Bev-VTE treatment group, 61.54% received prophylactic doses, and 38.46% received therapeutic doses.

Notably, the median duration of concurrent treatment was shorter for the VTE prophylaxis groups than the VTE treatment groups. Specifically, the median duration was 4 days in the TKI-VTE prophylaxis group and 28.5 days in the Bev-VTE prophylaxis group, whereas it was extended to 30 days in the TKI-VTE treatment group and 50 days in the Bev-VTE treatment group.

#### 3.1.2 Bleeding risk of concurrent therapy

**FIGURE 2:**
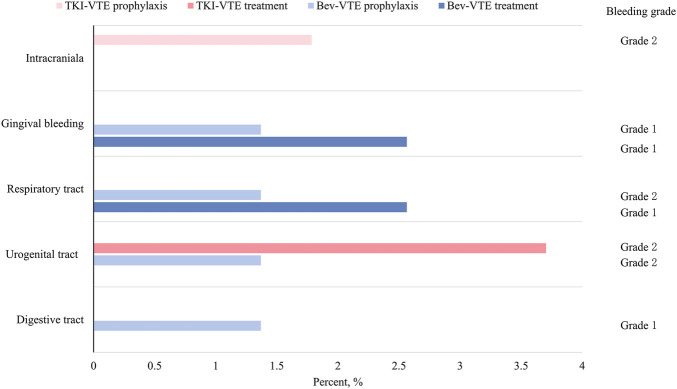
Bleeding sites, grades, and incidence in patients on concurrent anti-VEGF agents and anticoagulants in the retrospective analysis. The percentage of bleeding events at distinct anatomical sites for four groups: TKI-VTE prophylaxis, TKI-VTE treatment, Bev-VTE prophylaxis, and Bev-VTE treatment. Bleeding severity was graded according to CTCAE v5.0. Only one grade 2 intracranial bleeding event was clinically attributed to expected perioperative changes.

The incidence of bleeding events was low in patients receiving Bev- or TKI-based combination therapy (Figure 2). Notably, no treatment-related serious bleeding events (grade ≥3) were recorded across all groups. Merely one mild intracranial bleeding event (graded as 2) was detected via postoperative magnetic resonance imaging (MRI) and clinically attributed to anticipated surgical changes. The common bleeding sites included urogenital, gingival, and respiratory tract and gastrointestinal regions.

#### 3.1.3 The outcomes of thromboembolic events

**FIGURE 3:**
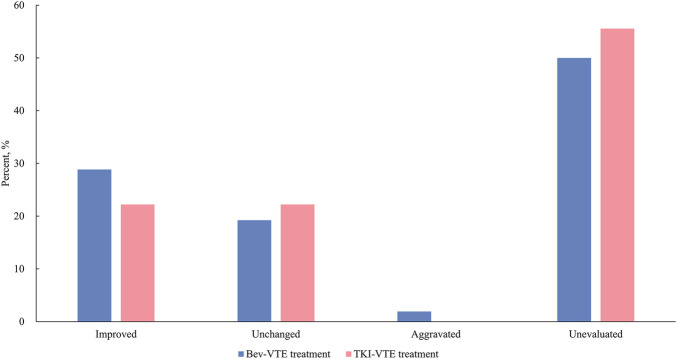
VTE outcomes in patients receiving concurrent anti-VEGF agents and anticoagulants in the retrospective analysis.

In our study, nearly a half of thrombosis cases did not undergo follow-up evaluation via B-ultrasound or CT (Figure 3). In the Bev-VTE treatment cohort, 38.46% of thrombosis demonstrated improvement, 25.46% remained stable, and 2.56% exhibited progression. In the TKI-VTE treatment cohort, 22.22% of thrombosis showed improvement, while a further 22.22% remained unchanged.

### 3.2 A pharmacovigilance analysis

#### 3.2.1 Descriptive analysis

A total of 1,138 bleeding events associated with the co-administration were reported, including 450 cases in the TKI-DOAC group, 233 in the TKI-LMWH group, 237 in the Bev-DOAC group, and 218 in the Bev-LMWH group. The characteristics of the bleeding reports are detailed in Table 2. Overall, more bleeding events were reported in male patients than in female patients. The median age of patients reporting bleeding events was 65 years old in the TKI-DOAC group, 64 years old in the TKI-LMWH group, 67 years old in the Bev-DOAC group, and 59 years old in the Bev-LMWH group. Most reports were from United States, followed by France and Canada. Cabozantinib and sunitinib were the most commonly reported TKIs. Rivaroxaban and apixaban were the most commonly reported DOACs. The indications for anticoagulants included AF, VTE prophylaxis, and VTE treatment. Among these indications, VTE treatment was recorded in 90 cases (20.00%) in the TKI-DOAC group, 80 (34.33%) in the TKI-LMWH group, 85 (35.86%) in the Bev-DOAC group, and 55 (25.23%) in the Bev-LMWH group.

**TABLE 2:** Characteristics of bleeding reports co-administrated with anti-VEGF agents and anticoagulants in FAERS.

Parameters	TKI-DOAC (n = 450)	TKI-LMWH (n = 233)	Bev-DOAC (n = 237)	Bev-LMWH (n = 218)
Female (n, %)^a^	144 (32.00%)	77 (33.05%)	100 (42.19%)	109 (50.00%)
Male (n, %)	288 (64.00%)	138 (59.23%)	126 (53.16%)	97 (44.50%)
Not specifed (n, %)	18 (4.00%)	18 (7.73%)	11 (4.64%)	12 (5.50%)
Median age (y, IQR)	65 (51–73)	64 (54–68)	67 (56–76)	59 (50–70)
TKI types (n, %)				
Cabozantinib	162 (36%)	50 (21.46%)	​	​
Sunitinib	89 (19.78%)	56 (24.03%)	​	​
Axitinib	61 (13.56%)	26 (11.16%)	​	​
Pazopanib	42 (9.33%)	36 (15.45%)	​	​
Lenvatinib	50 (11.11%)	28 (12.02%)	​	​
Regorafenib	43 (9.56%)	19 (8.15%)	​	​
Sorafenib	31 (6.89%)	16 (6.87%)	​	​
DOAC types (n, %)				
Rivaroxaban	194 (43.11%)	​	114 (48.10%)	​
Apaixaban	235 (52.22%)	​	153 (64.56%)	​
Dabigatran	13 (2.89%)	​	12 (5.06%)	​
Edoxaban	15 (3.33%)	​	28 (11.81%)	​
Tumour sites (n, %)				
Renal	250 (55.56%)	112 (48.07%)	​	​
Colorectal	36 (8.00%)	18 (7.73%)	67 (28.27%)	47 (21.56%)
Hepatic	31 (6.89%)	3 (1.29%)	21 (8.86%)	16 (7.34%)
Lung	​	​	37 (15.61%)	29 (13.30%)
Ovarian/Cervix	​	​	16 (6.75%)	39 (17.89%)
Glioblastoma	​	​	12 (5.06%)	30 (13.76%)
Other/Unknown	133 (29.55%)	100 (42.91%)	84 (35.45%)	57 (26.15%)
Anticoagulation indication (n, %)				
AF, VTE prophylaxis	53 (11.78%)	33 (14.16%)	63 (26.58%)	10 (4.59%)
VTE treatment	90 (20.00%)	80 (34.33%)	85 (35.86%)	55 (25.23%)
Not specifed	307 (68.22%)	120 (51.50%)	89 (37.55%)	153 (70.18%)
Report country (n, %)				
United States	321 (71.33%)	104 (44.64%)	79 (33.33%)	51 (23.39%)
France	23 (5.11%)	47 (20.17%)	15 (6.33%)	32 (14.68%)
Canada	15 (3.33%)	15 (6.44%)	29 (12.24%)	38 (17.43%)
Other/unknown	91 (20.22%)	67 (28.76%)	114 (48.10%)	97 (44.50%)
Outcome (n, %)				
DE	70 (15.56%)	56 (24.03%)	51 (21.52%)	85 (38.99%)
LT	22 (4.89%)	26 (11.16%)	19 (8.02%)	14 (6.42%)
DS	9 (2.00%)	4 (1.72%)	4 (1.69%)	-
HO	206 (45.78%)	85 (36.48%)	91 (38.40%)	41 (18.81%)
OT	88 (19.56%)	41 (17.60%)	59 (24.89%)	45 (20.64%)
Not specifed	55 (12.22%)	11 (4.72%)	13 (5.49%)	33 (15.14%)

AF, atrial fibrillation; DE, death; LT, Life-Threatening; DS, disability; HO, Hospitalization (or Prolonged Hospitalization); OT, Other Serious Important Medical Event.

^a^ All percentages were calculated relative to the respective column totals.

**FIGURE 4:**
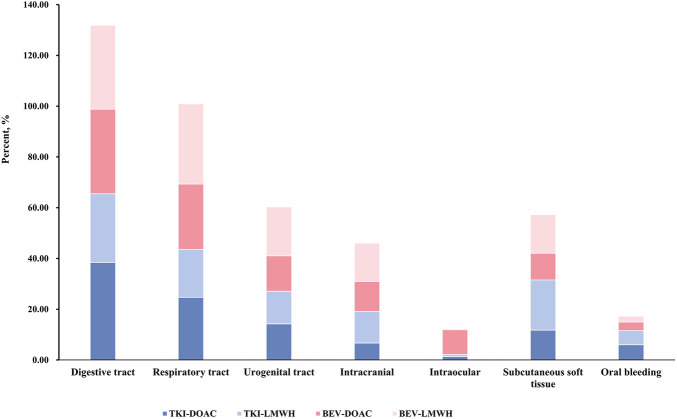
Bleeding sites and incidence of concomitant anti-VEGF agent and anticoagulant use in the pharmacovigilance analysis.

Bleeding events in the TKI-DOAC/TKI-LMWH groups occurred predominantly in RCC patients (53%), whereas those in the Bev-DOAC/Bev-LMWH groups were more common in colorectal cancer patients (25.05%). Bleeding sites were similar across groups, with the digestive tract, respiratory tract, urogenital tract, and subcutaneous tissue being the most common. Intracranial and intraocular hemorrhages were less frequent, as illustrated in Figure 4. Epistaxis (20.56%) was most commonly reported PT, followed by gastrointestinal hemorrhage (9.58%) and hemorrhage (9.40%), as shown in Supplementary Table S7.

#### 3.2.2 DDI of Anti-VEGF agents and anticoagulants

As shown in Table 3, Ω_025_ for the Bev-LMWH group was 0.06, indicating a synergistic increase in bleeding risk. In contrast, this value was negative for the TKI-DOAC, TKI-LMWH and Bev-DOAC groups, suggesting no elevated bleeding risk with these combinations.

**TABLE 3:** Shrinkage analysis for anti-VEGF agents and anticoagulants interaction.

Drug1	Drug2	Ω	Ω_025_
TKI	DOAC	−0.92	−1.05
	LMWH	0.10	−0.09
Bev	DOAC	−0.38	−0.57
	LMWH	0.25	0.06

## 4 Discussion

The concurrent use of anti-VEGF agents and anticoagulants has become increasingly inevitable in oncology, yet the associated bleeding risks remain largely elusive. To address this, we conducted a dual-approach study integrating a retrospective and a disproportionality analysis. To our knowledge, this is the most comprehensive retrospective analysis of the concurrent use of anti-VEGF agents and anticoagulants to date. Meanwhile, we are the first to use FAERS to assess bleeding risks of various anti-VEGF-anticoagulant combinations, providing more real-world evidence on DDIs.

Our institutional cohort was characterized by a short median overlap between anti-VEGF agents and anticoagulants, along with a predominance of prophylactic-dose anticoagulation. This may be explained as follows: Reduced-dose anticoagulation was associated with fewer bleeding complications compared with full-dose anticoagulation (Mahé et al., 2025). For VTE treatment, particularly in the Bev subgroup, most thrombotic events were identified as non-acute, non-proximal DVTs. Given this clinical characteristic, most clinicians tended to adopt a conservative anticoagulant dose to balance the risk of thromboembolism and bleeding. For VTE prophylaxis, clinicians focused on reducing bleeding risk, which accounted for the short overlap.

The short median treatment overlap and the predominance of prophylactic-dose anticoagulation likely contributed to the low bleeding event incidence observed in the present study. Additionally, patients with RCC—a population with inherently elevated bleeding risk—were underrepresented in our cohort. Furthermore, evidence has confirmed that bleeding risk varies across different TKI agents (Lee et al., 2023). A network meta-analysis of 11 TKIs revealed that sunitinib and regorafenib were associated with an increased risk of bleeding incidence (Das et al., 2021). Notably, the TKIs included in our study were primarily apatinib, lenvatinib, and regorafenib. Collectively, these factors may account for the low incidence of bleeding events observed in the present study.

While several studies have examined the co-administration of TKIs and anticoagulants, the available evidence is inconsistent. For example, one prior study reported the safe concurrent administration of therapeutic DOACs with TKIs even in a cohort comprising 48% RCC patients (Boileve et al., 2023). However, this study did not clarify the types of TKIs included, which might compromise the generalizability of the findings. Heterogeneity in the definition of bleeding endpoints may also explain discrepancies across study outcomes. For instance, when bleeding endpoints were defined as clinically significant bleeding events (encompassing major bleeding and clinically relevant non-major bleeding [CRNMB]), TKI combined with therapeutic-dose LMWH was associated with an increased bleeding risk (Patel et al., 2021). In contrast, when the endpoint was restricted to major bleeding alone, TKIs (n = 74) combined with therapeutic-dose DOACs (50%) and LMWH (20%) did not elevate the risk of major bleeding events (Sivakumar et al., 2021).

The concurrent administration of Bev and anticoagulants is rarely involved in the existing literature. Our findings demonstrated that the short-term concurrent use of Bev with rivaroxaban—particularly at prophylactic doses—was safe. Consistent with our results, several small-scale studies reported favorable safety profiles for Bev-DOAC combination in ovarian cancer (n = 7; 4 cases on apixaban, 2 on edoxaban, and 1 on rivaroxaban) and Bev-apixaban combination in colorectal cancer (n = 16) (Komiyama et al., 2019; Rho et al., 2023). Conversely, one study documented that the incidence of grade 3 bleeding events was 13% among patients treated with Bev plus DOACs (33% apixaban, 67% rivaroxaban). Emerging evidence further suggests that bleeding risks may differ across different DOACs used in combination with Bev. For example, a study previous regarding colorectal cancer revealed that patients treated with Bev plus apixaban had a lower incidence of bleeding complications than those receiving Bev plus rivaroxaban (Rho et al., 2023).

The FAERS encompassed a broader spectrum of combination therapy scenarios, including a higher proportion of RCC patients (nearly 50% in the TKI cohort), a more diverse range of anticoagulants (rivaroxaban, apixaban and LMWH), and seven TKIs subtypes (including sunitinib and regorafenib). These might capture higher-risk scenarios, such as Bev-LMWH, which contributed to addressing the paucity of real-world data in our retrospective analysis. Bleeding events associated with BEV-LMWH co-administration have also been reported in clinical cases. For instance, a lung cancer patient developed clinically significant lumbosacral hematomas within 1 week of initiating Bev plus LMWH therapy (Munoz et al., 2012). Notably, due to the spontaneous reporting characteristic of FAERS, the safety signals for TKI-anticoagulant and Bev-DOAC combinations do not guarantee their absolute safety in clinical practice. Vigilant monitoring is still required for high bleeding-risk patients (e.g., RCC) and high bleeding-risk drugs (e.g.,,sunitinib, regorafenib). Similarly, the weak signal associated with Bev-LMWH combinations dose not preclude their clinical use.

Mechanistically, anti-VEGF agents increase bleeding risk by disrupting the endothelial barrier, thrombocytopenia, and platelet dysfunction, while anticoagulants exacerbate bleeding by interfering with coagulation cascades and fibrinolysis (Watson and Al-Samkari, 2021; Lee et al., 2025; Sobolewska et al., 2021). The co-administration of anti-VEGF agents and anticoagulants may produce a synergistic effect, athough further validation is required. A mechanistic study showed that heparin regulated the biological activity of VEGF by binding to VEGF165, the predominant human VEGF isoform, thereby further disrupting endothelial cell function and angiogenic signaling (Nemashkalova et al., 2023). This interaction may increase bleeding risk when heparin-based anticoagulants are combined with anti-VEGF agents. For instance, a phase I trial involving the combination of sunitinib and dalteparin in 17 RCC patients reported an elevation of anti-factor Xa levels and prolongation of activated partial thromboplastin time (aPTT) (Wentink et al., 2017), in accordance with a more pronounced signal for the Bev-LMWH combination in FAERS.

However, several limitations must be acknowledged within our retrospective analysis. Firstly, the retrospective design has some inherent biases including the accuracy and integrity of data. For instance, mild-to-moderate bleeding events might be underreported in EMRs, which could introduce bias into bleeding risk assessment. Limited follow-up imaging data for thrombotic cases also restricted the ability to fully interpret the efficacy of the anticoagulant regimens. Furthermore, our study involved a diverse group of patients with different characteristics, which might influence bleeding risk during concurrent treatment. However, the small sample size limited our ability to conduct subgroup analyses based on cancer types, TKI subtypes, DOAC subtypes, and other clinical factors.

The FAERS analysis also had inherent limitations. Firstly, as a spontaneous, anonymous reporting system, it inherently sufferd from underreporting, overreporting, and incomplete data. Secondly, the lack of critical data (e.g., anticoagulant dosage, precise treatment timelines) impeded in-depth analysis, and the lack of patient-related information (e.g., comorbidities) might be a confounding factor affecting the results. Furthermore, this analysis could neither establish a causal relationship between bleeding events and drug combinations, nor calculate the corresponding bleeding rates. Despite these limitations, pharmacovigilance analysis serves as a cornerstone for studying adverse drug reactions and DDIs.

In conclusion, TKI-DOAC/LMWH and Bev-DOAC co-administration appear to be safe, especially with short-term, prophylactic anticoagulant dosages. While a potential bleeding signal was observed for Bev-LMWH co-administration, highlighting the necessity for careful clinical monitoring during concurrent use. As this study is exploratory and hypothesis-generating, it cannot establish definitive safety profiles or causal relationships between these regimens and bleeding events. Therefore, further prospective controlled studies with standardized anticoagulant dosing, systematic bleeding risk assessment, and stratified subgroup analysis are warranted to validate these findings.

## Data Availability

The original contributions presented in the study are included in the article/Supplementary Material, further inquiries can be directed to the corresponding author.
