# Supplementary material for: Exploratory assessment of bleeding risk associated with concurrent use of Anti-VEGF agents and anticoagulants in oncology
Source: Front Pharmacol. 2026 Apr 8;17:1759971. doi: 10.3389/fphar.2026.1759971 (PMC13099126; doi:10.3389/fphar.2026.1759971)
Supplement: Supplementary file 1 [file DataSheet2.doc]

Supplementary Material

# **1 Supplementary Figures**


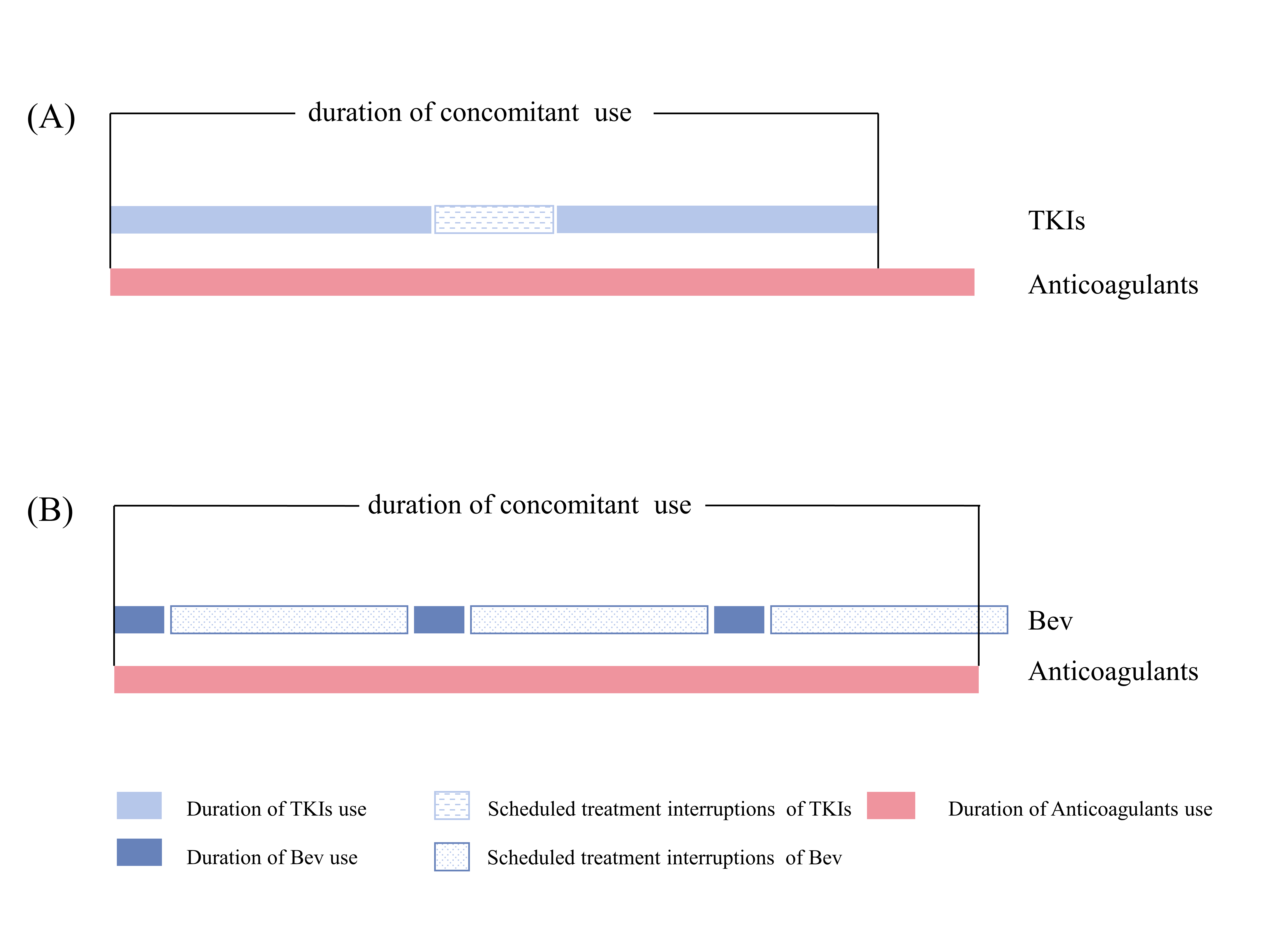


Figure S1 Calculation Method for Concomitant Duration of Bev/TKIs and Anticoagulant Administration

**2 Supplementary Tables**

Table S1 Categorization of anticoagulant dosages in our study

| **Anticoagulants** | **Prophylactic** | **Intermediate** | **Therapeutic** |
| --- | --- | --- | --- |
| Rivaroxaban | 10mg qd |  | 15mg bid/20mg qd |
| Enoxaparin | 4000 iu qd or less | Between prophylactic dosage and therapeutic dosage | 100 iu/kg q12h or 150 iu/kg qd |
| Nadroparin | 4100 iu qd or less | Between prophylactic dosage and therapeutic dosage | 95 iu/kg q12h |
| LMWH | 4000 iu qd or less | Between prophylactic dosage and therapeutic dosage | 100 iu/kg q12h |

Table S2 The generic/ brand names of anti-VEGF agents and anticoagulants

| **Drug category** | **Generic names** | **Brand names** |
| --- | --- | --- |
| Anti-VEGF mAb | Bevacizumab | Abevmy, Alymsys, Avastin, Avzivi, Aybintio, Lytenava, Mvasi, Oyavas, Vegzelma, Zirabev |
| Anti-VEGF TKI | Sorafenib | Nexavar, Sorafenib Accord |
|  | Sunitinib | Sunitinib Accord, Sutent |
|  | Pazopanib | Votrient |
|  | Axitinib | Inlyta |
|  | Cabozantinib | Cabometyx, Cometriq |
|  | Lenvatinib | Kisplyx, Lenvima10 |
|  | Regorafenib | Stivarga |
|  | Vandetanib | Caprelsa |
| DOAC | Rivaroxaban | Rivaroxaban Mylan,Xarelto |
|  | Apixaban | Eliquis |
|  | Dabigatran | Dabigatran etexilate, Pradaxa |
|  | Edoxaban | Edoxaban mesylate, Edoxaban tosylate, Lixiana, Roteas, Savaysa |
| LMWH | Dalteparin | Fragmin |
|  | Enoxaparin | Inhixa,Lovenox,clexane |
|  | Nadroparin | Fraxiparine,fluxum |
|  | Bemiparin | Hibor |
|  | Parnaparin |  |
|  | Reviparin |  |
|  | Clivarina | Danaparoid |
|  | Tinzaparin | Innohep, Sulodexide, Bemiparin |
|  | Low molecular weight heparin | LMWH |

Table S4 The 4 × 2 contingency table for calculation of DDI signal, Ω

|  | **Target AE** | **Other AEs** | **Total** |
| --- | --- | --- | --- |
| Concomitant use of drug D1 and drug D2 | n111 | n110 | n11+ |
| drug D1 without drug D2 | n101 | n100 | n10+ |
| drug D2 without drug D1 | n011 | n010 | n01+ |
| Neither drug D1 nor drug D2 | n001 | n000 | n00+ |
| Total | n++1 | n++0 | n+++ |

AE: adverse event, *n*: the number of reports.


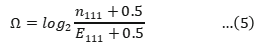


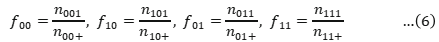


Where, *n* is the number of reports shown in the 4 × 2 contingency table.


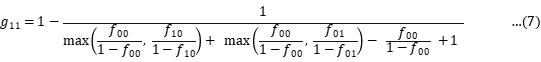


When *f*_10_ < *f*_00_ (which denote no risk of AE caused by *drug D*_1_), the most sensible estimator *g*_11_ = max (*f*_00_, *f*_01_) is yielded and the *vice versa* when *f*_01_ < *f*_00_.


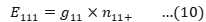


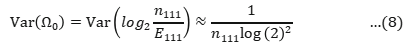


Where, *n*_111_ is the number of reports and *E*_111_ is the expected value.


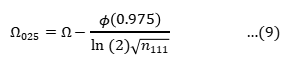


Where, *ϕ* (0.975) is 97.5% of the standard normal distribution.

Table S5 Distribution of Thrombosis Sites and Acute/Non-Acute Status in Patients Treated with TKIs and Bev

| Group | Thrombosis Site  (n,%) | Acute Thrombosis (n,%) | Non-acute Thrombosis (n,%) |
| --- | --- | --- | --- |
| TKI-VTE treatment | Distal/ Upper Extremity DVT | 7 (26%) | 4 (15%) |
|  | Proximal DVT | 6 (22%) | 0 (0%) |
|  | Visceral DVT | 2 (7%) | 2 (7%) |
|  | PE | 4 (15%) | 2 (7%) |
| Bev-VTE treatment | Distal/ Upper Extremity DVT | 7 (13%) | 40 (77%) |
|  | Proximal DVT | 1 (2%) | 2 (4%) |
|  | Visceral DVT | 0 (0%) | 0 (0%) |
|  | PE | 0 (0%) | 2 (4%) |

Table S6 Baseline Factors Influencing Bleeding Risk in the Study Cohort

| Parameters | TKI-VTE prophylaxis  （n=56） | TKI-VTE  treatment  （n=27） | Bev-VTE prophylaxis  （n=73） | Bev-VTE  treatment  （n=52） |
| --- | --- | --- | --- | --- |
| Grade 1 thrombocytopenia (n,%) | 1 (2%) | 1 (4%) | 1 (1%) | 1 (2%) |
| Grade 2 thrombocytopenia (n,%) | 2 (4%) | 1 (4%) | 2 (2%) |  |
| Crcl 45-59mL/min (n,%) | 3 (5%) | 1 (4%) | 1 (1%) | 2 (4%) |
| CTP A (n,%) | 2 (4%) | - | - | 1 (2%) |
| CTP B (n,%) | - | 2 (8%) | - | - |
| Brain metastases (n,%) | - | - | - | 1 (2%) |

CTP: Child-Turcotte-Pugh; Crcl:Creatinine Clearance;

Table S7 PTs of hemorrhagic events in patients co-administered anti-VEGF agents and anticoagulants

| **PT** | **Bev-DOAC**  **(%)** | **Bev-LMWH**  **(%)** | **TKI-DOAC**  **(%)** | **TKI-LMWH (%)** |
| --- | --- | --- | --- | --- |
| Epistaxis | 21.94 | 28.44 | 20.00 | 12.86 |
| Gastrointestinal haemorrhage | 8.44 | 9.17 | 11.56 | 7.30 |
| Haemorrhage | 7.17 | 9.17 | 11.11 | 8.58 |
| Contusion | 6.75 | 6.88 | 7.56 |  |
| Haematochezia | 9.28 | 11.92 | 6.89 | 5.58 |
| Haematuria | 8.02 | 5.50 | 5.78 |  |
| Haematoma |  |  |  | 7.72 |
| Haemoptysis |  |  |  | 5.58 |
| Others | 38.4 | 28.92 | 37.11 | 52.38 |
